# Assessing the locomotor demands of international men’s and women’s rugby sevens match-play according to passage of play

**DOI:** 10.1371/journal.pone.0304186

**Published:** 2024-06-14

**Authors:** Samuel P. Hills, Erwan Izri, Daniel Howells, Ben Lonergan, Liam P. Kilduff, Mark Waldron

**Affiliations:** 1 Faculty of Health and Social Sciences, Bournemouth University, Bournemouth, United Kingdom; 2 Applied Sports Technology, Exercise Medicine Research Centre (A-STEM), Swansea University, Swansea, United Kingdom; 3 School of Sport and Health Sciences, University of Brighton, Eastbourne, United Kingdom; 4 Chelsea Football Club, Cobham, Surrey, United Kingdom; 5 Welsh Institute of Performance Science, College of Engineering, Swansea University, Swansea, United Kingdom; 6 School of Health and Behavioural Sciences, University of the Sunshine Coast, Sippy Downs, Queensland, Australia; Tokat Gaziosmanpasa University Tasliciftlik Campus: Tokat Gaziosmanpasa Universitesi, TURKEY

## Abstract

This study aimed to evaluate the effect of discrete passages of play on locomotor demands of international men’s and women’s rugby sevens matches and their relationship with winning or losing. Thirteen men’s and thirteen women’s international rugby sevens players wore 10 Hz Global Positioning Systems during twelve Tokyo Olympic games matches (966 observations; 507 for men, 459 for women). Discrete ball-in-play periods were categorised as: ‘Single-phase defence’, ‘single-phase attack’, ‘multi-phase defence’, ‘multi-phase attack’, ‘multi-phase defence to attack’, or ‘multi-phase attack to defence’. Relative total distance, alongside high-speed (>5.0 m∙s^-1^), acceleration (>3 m∙s^-2^), and deceleration (>3 m∙s^-2^) distances were recorded for each passage. Separately for men and women, linear mixed models examined the effect of passage type and match outcome (win or loss) on locomotor demands, whilst controlling for opposition ranking. In men, relative total distance ranged from 137 m∙min^-1^ to 174 m∙min^-1^ for ‘multi-phase defence to attack’ and ‘multi-phase attack’, respectively. In women, ‘multi-phase attack’ elicited the lowest relative total distance (118 m∙min^-1^), whereas the greatest values (186 m∙min^-1^) were recorded for ‘single-phase defence’. For men, there were significant interactions between match outcome and passage type for relative total (p<0.001) and high-speed (p = 0.006) distance. During ‘multi-phase attack’, relative total distance was greater for wins versus losses (174 vs 138 m.min^-1^, p = 0.024). However, for ‘single-phase defence’, relative total distance was lower for wins (128 vs 164 m.min^-1^, p<0.001). For women, there were significant interactions between match outcome and passage type for relative total (p = 0.036), high-speed (p = 0.003), and deceleration (p = 0.015) distances. Locomotor responses were influenced by passage type and match result for men and women. Knowing the demands of each passage type may inform training drills targeted at developing match-play-specific physical, technical, and tactical adaptations. Understanding how passages differ between matches won and lost could also inform team technical/tactical preparation including selection.

## Introduction

Rugby sevens involves two teams of seven players competing in matches contested over seven min halves, separated by a two min half-time. Teams competing at the Tokyo 2020 Olympics completed between three and six matches within 72 h. International men’s players typically cover approximately 91 to 120 m∙min^-1^ across a whole-match [1–3], with 17 to 19% of total distance (TD) being accumulated at speeds more than 5 m∙s^-1^ (high-speed running; HSR) [1,4]. International women’s players may achieve broadly similar average speeds of 85 to 101 m∙min^-1^ [5–7], but potential differences in physiological characteristics and/or playing styles mean that HSR comprises just 6 to 13% of TD when the same 5 m∙s^-1^ threshold is used [5–7].

The stochastic nature of rugby sevens means that a player’s physical load is not accumulated linearly throughout a fixture [8–10]. Although whole-match activity profiles are useful when evaluating the overall physical loads that players experience during match-play, it is important that players are conditioned to tolerate the elevated physical demands associated with certain phases within a match. Indeed, the ball may be live for less than 50% of the total match duration and more than 90% of ball-in-play periods last less than 60 s [11]. As such, quantification of the ‘peak’ or ‘worst case scenario’ (WCS) locomotor demands of international rugby sevens match-play is now commonplace [12–14]. For example, the most demanding 60 s period of international rugby sevens matches may require men’s players to achieve approximately 173 to 184 m of TD or 64 m of HSR, which is substantially different from the average demands of more prolonged match periods [10,13,14].

Whilst WCS can be used to develop training drills that reflect the most demanding passages of match-play, a univariate approach to WCS quantification does not provide any additional information as to the circumstances in which such demands were experienced. Matches comprise distinct passages of play, which represent ‘ball-in-play’ periods. These passages can involve either a single-phase or comprise multiple phases, during which a team may be attacking, defending, or transitioning from one to the other. As each of these passage types (i.e., single or multi-phase; attack, defence, or transition) may require execution of distinct movement patterns and tactical strategies, coaching in rugby sevens often involves understanding the behaviours inherent within each passage type.

In any team sport, the aim of a given passage is likely to affect the physical demands that both teams experience within that period of play. Elite soccer forwards experienced greater peak demands when their team was in possession of the ball, whilst this relationship was reversed for defenders [15]. Similarly, field position and passage aims have influenced physical responses in professional Australian Football [16]. Quantifying the demands associated with different passage types during wins and losses would provide valuable reference values for international men’s and women’s rugby sevens [17], and could inform the design and monitoring of training drills that seek to prepare players for the physical, technical, and tactical demands of match-play. Indeed, the concept of ‘tactical periodisation’ involves coaching staff contributing to the co-design of training activities, with the aim of targeting simultaneous development of several key elements of the sport [18]. Thus, the aim of this study was to evaluate the effect of discrete single and multi-phase attacking, defending and transition passages on locomotor demands in international men’s and women’s rugby sevens and their relationship with match outcome (won or lost), whilst controlling for the ranking of the opposition team. It was hypothesised that passage type and match outcome would each affect locomotor responses for both men’s and women’s players.

## Materials and methods

Following ethical approval from the XXXXXXXXXXXXXX Ethics Committee, players from international men’s (*n* = 13, age: 29 ± 3 years, stature: 180 ± 6 cm, mass: 89 ± 7 kg) and women’s (*n* = 13, age: 25 ± 5 years, stature: 175 ± 4 cm, mass: 73 ± 4 kg) rugby sevens squads were monitored during the Tokyo 2020 Olympics tournaments. Participants provided written informed consent before data collection. From twelve matches (six men’s, six women’s), 966 individual player observations were yielded (nOBS = 507, 3–66 observations per player; and nOBS = 459, 1–69 observations per player for men’s and women’s, respectively). Data were included from all players involved in any given passage.

Players were monitored via Microelectromechanical Systems devices containing Global Positioning Systems (GPS; 10 Hz; STATSport, Northern Ireland), which were housed between the scapulae in a pocket designed to minimise movement artifact. Such technology has shown good reliability (coefficients of variation: 0.3% and 1.3% for assessment of TD and HSR, respectively) [19] and validity (mean bias: <2.5%) for measuring distances during team sports movements [20]. Players were familiar with this form of activity profiling and each individual wore the same unit throughout to avoid the influence of inter-unit variation. Devices were activated before each pre-match warm-up according to the manufacturer’s guidelines, and data were downloaded after each match using proprietary software (Apex Rugby, Team Series, STATSports). For all files, horizontal dilution of position was <1.5 and the number of satellite connections was >8. Matches were also filmed by the lead analyst for the relevant team, before this footage was overlayed and synchronised with GPS data using STATSports Apex software.

Total ball-in-play time for each match was divided into distinct passages of play (i.e., from the ball becoming live until the next stoppage) by the lead performance coaches. Passages were categorised as either: 1) ‘Single-phase defence’; 2) ‘Single-phase attack’; 3) ‘Multi-phase defence’; 4) ‘Multi-phase attack’; 5) ‘Multi-phase defence to attack’; or 6) ‘Multi-phase attack to defence’.

- *Single-phase refers to a period for which the ball was in play and no breakdowns (e*.*g*., *tackling*, *ruck forming*, *etc) occurred*.
- *Multi-phase refers to a period for which the ball was in play and where one or more breakdowns occurred*.
- *Transition passages (i*.*e*., *categories 5 and 6 above) involved a change of possession within the passage*, *whereby ‘multi-phase attack to defence’ involved the reference team gaining possession from the opposition during the passage*, *whilst ‘multi-phase attack to defence’ involved the reference team losing possession having begun the passage with possession*.

A single experienced analyst classified all passages according using the ball becoming “live” and becoming “dead” as start and end points for a given passage, respectively. Match outcome was coded as either a ‘win’ or ‘loss’ according to the final result of each match. Opposition ranking was determined based on whether the reference team’s official world ranking was ‘above’ or ‘below’ that of the opposition team.

The four GPS-derived locomotor variables of interest were: TD (distance accumulated per passage at any speed), HSR (distance covered per passage at speeds >5 m∙s^-1^), high-speed acceleration distance (distance covered per passage whilst accelerating at a rate >3 m∙s^-2^), and high-speed deceleration distance (distance covered per passage whilst decelerating at a rate >3 m∙s^-2^). All variables were expressed relative to passage length (i.e., m∙min^-1^) to account for the natural variation in phase durations. These variables reflect those that were of interest to performance staff working with the team to aid training prescription and monitoring during technical/tactical drills that are targeted at the physical demands of match-play. Given the limited number of total breakdowns in rugby sevens matches, the focus of this study was on locomotor variables.

### Statistical analyses

All data were log-transformed and within-player centred (i.e., each individual’s datum was expressed relative to the mean of the same individual’s data) prior to analysis [21]. Linear mixed models were performed on men’s and women’s data separately using the *lmerTest* package in RStudio (Version 2022.07.1; RStudio, Boston, USA). To assess differences in locomotor demands between passages, passage type and match outcome were included as fixed factors, whilst opposition rank was included as a covariate. An interaction model was then constructed for all four dependent variables, which included passage type and match outcome as fixed factors, with opposition rank as a covariate. To account for the different number of observations contributed by each player to their respective datasets, player identification number was included as a random effect in all models. Model residuals were assessed for normality by visually observing the residuals Q-Q plots and performing Shapiro-Wilk normality tests. In the presence of significant (p <0.05) fixed effects or interactions, post-hoc tests with Bonferroni corrections were performed to identify pairwise differences within factors. Standardised effect sizes (d) were calculated using the *eff_size* and *emmeans* packages in RStudio, and were interpreted as: <0.20, *trivial*; 0.21–0.60, *small*; 0.61–1.20, *moderate*; 1.21–2.0, *large*; and >2.01, *very large* effects [22]. Visualisations were produced based on original non-transformed data using *dplyr* and *ggplot2* packages in RStudio.

## Results

Men’s and women’s teams each won three and lost three matches during the study period. Average passage durations are presented in Table 1, whilst Table 2 shows descriptive statistics and player-level coefficient of variation (CV) values for the four dependent variables (relative TD, HSR, high-speed acceleration and deceleration distances). CV ranged from 10% to 31% for the men, and 19% to 50% for the women.

**Table 1:** Passage and total time durations and number of observations across wins and losses for men and women. Data are presented as mean and standard deviation for durations (seconds) and the number of observations per category.

	Men								Women							
	Win				Loss				Win				Loss			
Passage type	Mean	SD	Total time	*nOBS*	Mean	SD	Total time	*nOBS*	Mean	SD	Total time	*nOBS*	Mean	SD	Total time	*nOBS*
Single phase defence	17	8	840	49	15	7	1064	70	23	18	821	36	16	0	96	6
Single phase attack	15	7	931	63	25	9	1393	56	10	0	70	7	10	3	529	53
Multi-phase defence	54	18	2632	49	56	39	2352	42	54	41	1559	29	30	16	3066	102
Multi-phase attack	38	12	2142	56	48	30	1304	27	41	16	2689	65	46	19	3116	68
Multi-phase defence to attack	60	23	2475	41	81	22	2178	27	55	31	2947	54	61	19	792	13
Multi-phase attack to defence	113	0	791	7	49	3	970	20	48	8	625	13	46	24	595	13
**Overall**	37	26	9811	265	38	30	9261	242	43	28	8711	204	32	21	8194	255

*nOBS*: Number of observations, SD: Standard deviation.

**Table 2:** Descriptive statistics for men’s and women’s data for each dependent variable.

	Men			Women		
Variable (relative)	Mean	SD	CV	Mean	SD	CV
**Total distance (m·min^-1^)**	160	16	10%	135	26	19%
**High-speed running distance (m·min^-1^)**	48	15	31%	31	12	38%
**High-speed acceleration distance (m·min^-1^)**	49	11	22%	36	11	31%
**High-speed deceleration distance (m·min^-1^)**	12	3	21%	10	3	34%

CV: Coefficient of variation, SD: Standard deviation.

There were significant main effects of passage type for TD, HSR, and deceleration for both men and women (all p <0.05). There were no significant main effects of match outcome on any dependent variable for men or women, except for HSR in the women’s data (p = 0.025).

**Fig 1:**
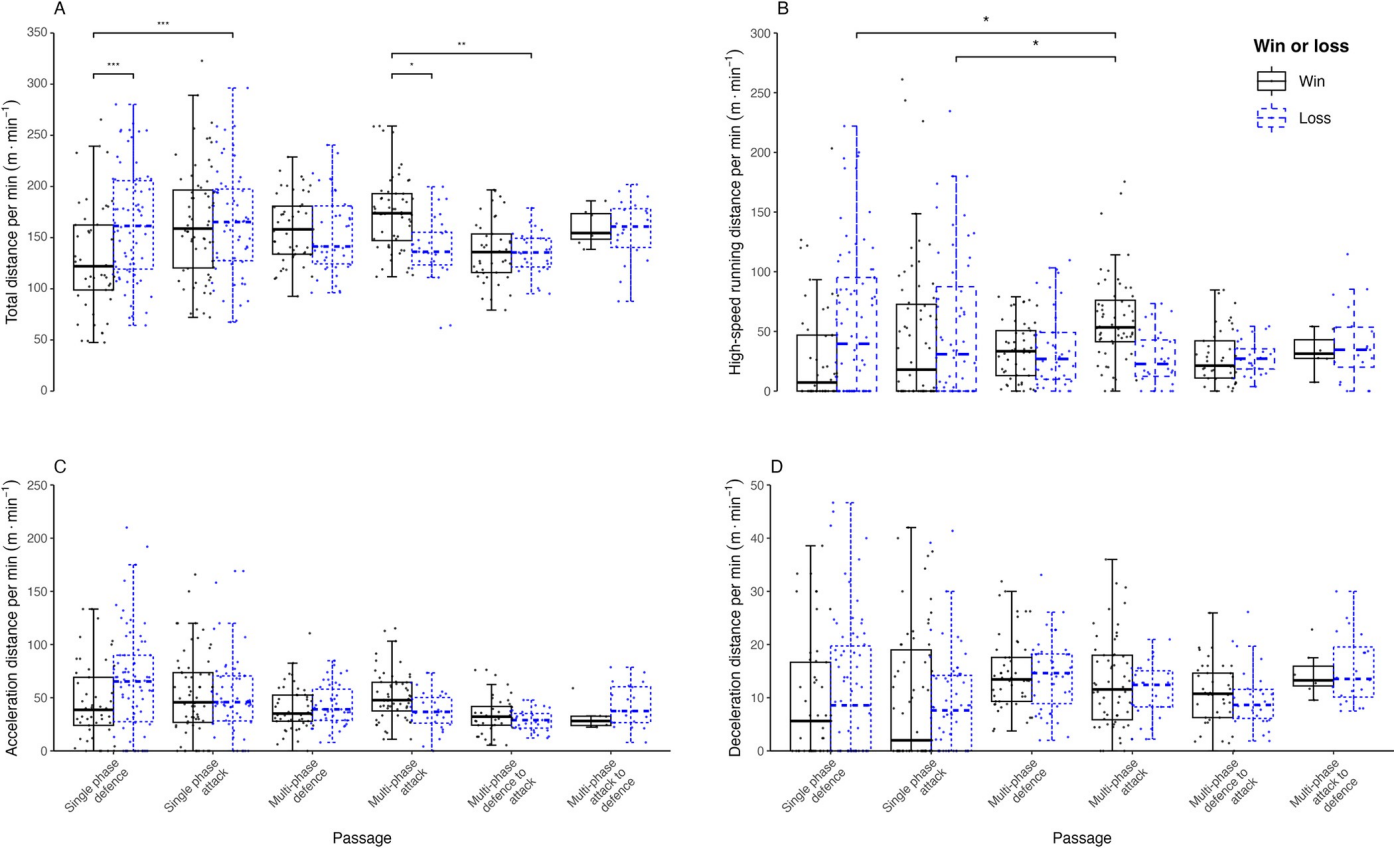
Men’s data for relative total distance (panel A), relative high-speed running distance (panel B), relative acceleration distance (panel C), and relative deceleration distance (panel D) for matches won (black) and lost (blue) across the six passage types. Data are presented as median values, upper and lower quartiles, and whisker boundaries representing 1.5 * inter-quartile range for each category. *, **, and *** denote significant pairwise contrasts at the p <0.05, p <0.01 and p <0.001 levels, respectively.

Fig 1 and Table 3 show that for men, there were significant interactions between match outcome and passage type for TD (p = 0.000) and HSR (p = 0.006), but not for acceleration (p = 0.380) or deceleration (p = 837). During ‘multi-phase attack’, TD was greater for wins versus losses (p = 0.024, d = 0.93, *moderate*). However, for ‘single phase defence’, per passage TD was lower for wins (p <0.001, d = 0.94, *moderate*). During losses, players covered more TD during ‘single phase attack’ compared with ‘single phase defence’ during wins (p <0.001, d = 0.96, *moderate*), whilst TD for ‘multi-phase attack’ during wins exceeded values recorded for ‘multi-phase defence to attack’ during losses (p = 0.010, d = 0.94, *moderate*). For HSR, values were greater during ‘multi-phase attack’ for wins, compared with both ‘single-phase defence’ during wins (p = 0.014, d = 0.75, *moderate*) and ‘single-phase attack’ during losses (p = 0.028, d = 0.72, *moderate)*.

**Table 3:** Locomotor demands across each passage type and win or loss for men and women.

		Men				Women			
		Win		Loss		Win		Loss	
Variable	Passage type	Mean	SD	Mean	SD	Mean	SD	Mean	SD
Total distance (m.min-1)	Single phase defence	128	53	164	54	170	57	186	29
	Single phase attack	165	55	165	53	169	32	147	71
	Multi-phase defence	158	33	154	38	124	29	169	48
	Multi-phase attack	174	36	138	32	135	36	118	27
	Multi-phase defence to attack	137	30	135	21	123	31	132	18
	Multi-phase attack to defence	160	17	157	30	157	49	138	25
High-speed running distance (m.min-1)	Single phase defence	30	45	57	63	60	69	86	38
	Single phase attack	46	61	53	61	45	45	45	72
	Multi-phase defence	34	24	36	33	16	20	44	46
	Multi-phase attack	60	37	28	23	25	28	13	18
	Multi-phase defence to attack	29	23	28	14	16	16	17	12
	Multi-phase attack to defence	33	16	39	31	39	32	12	9
High-speed acceleration distance (m.min-1)	Single phase defence	46	36	64	49	61	36	81	26
	Single phase attack	53	39	53	39	55	43	57	49
	Multi-phase defence	40	21	43	19	31	18	45	29
	Multi-phase attack	52	23	37	18	34	19	26	15
	Multi-phase defence to attack	34	17	29	10	27	15	32	11
	Multi-phase attack to defence	32	13	42	20	31	15	36	21
High-speed deceleration distance (m.min-1)	Single phase defence	10	11	11	13	12	11	18	4
	Single phase attack	10	12	9	10	18	13	10	14
	Multi-phase defence	15	7	14	7	11	7	11	8
	Multi-phase attack	13	9	11	5	10	8	9	6
	Multi-phase defence to attack	11	6	10	6	8	6	11	5
	Multi-phase attack to defence	15	4	15	7	10	7	12	7

SD: Standard deviation.

**Fig 2:**
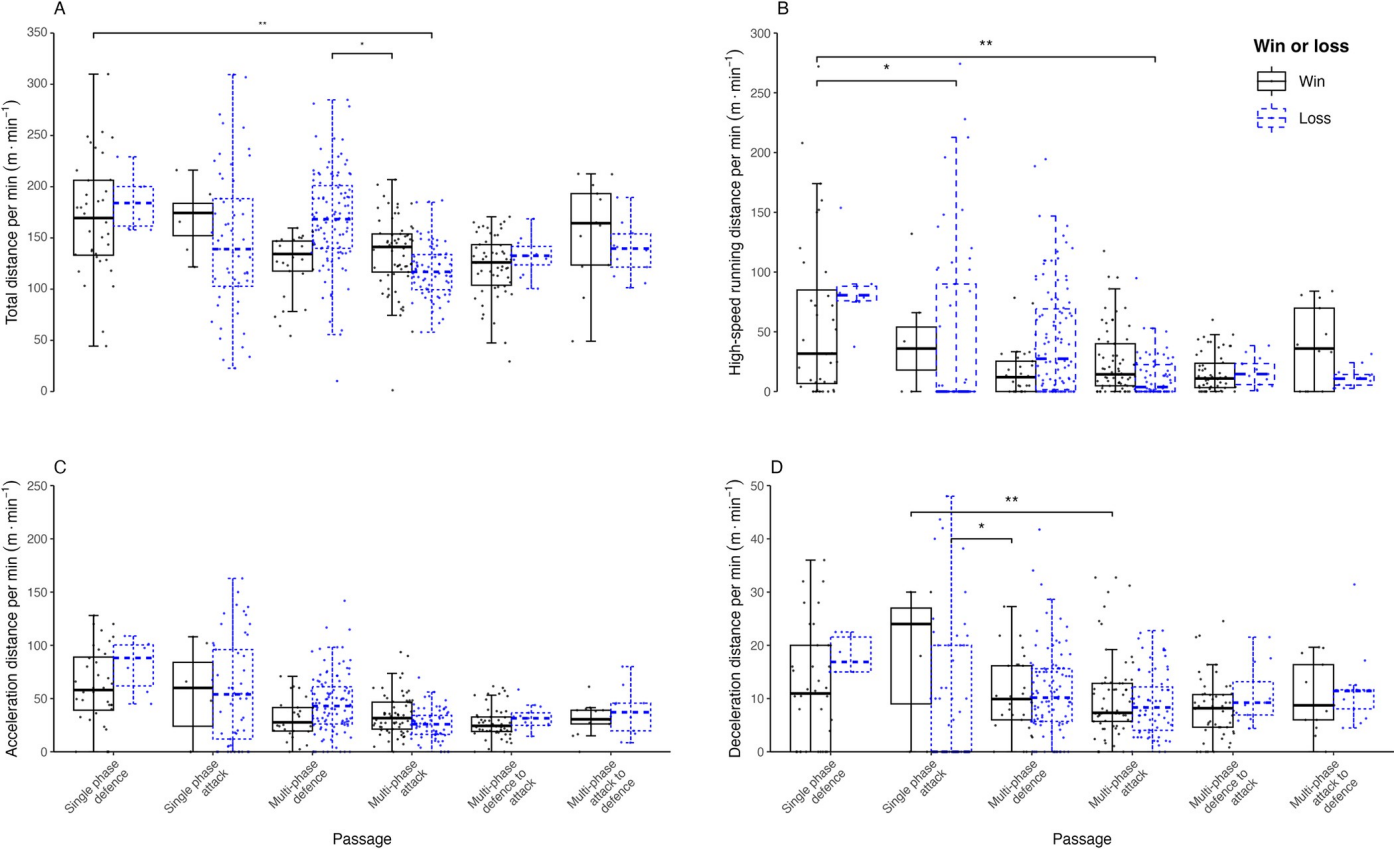
Women’s data for relative total distance (panel A), relative high-speed running distance (panel B), relative acceleration distance (panel C), and relative deceleration distance (panel D) for matches won (black) and lost (blue) across the six passage types. Data are presented as median values, upper and lower quartiles, and whisker boundaries representing 1.5 * inter-quartile range for each category. * and ** denote significant pairwise contrasts at the p <0.05 and p <0.01 levels, respectively.

Fig 2 and Table 3 show that for women, there were significant interactions between match outcome and passage type for TD (p = 0.036), HSR (p = 0.003), and deceleration (p = 0.015), but not for acceleration (p = 0.065). Greater TD was recorded in ‘single-phase defence’ during wins, compared with ‘multi-phase attack’ during losses (p = 0.003, d = 0.86, *moderate*). However, TD was lower for ‘multi-phase attack’ during wins, compared with ‘multi-phase defence’ during losses (p = 0.041, d = 0.57, *small*). For ‘single-phase defence’ during wins, HSR was greater than ‘single-phase attack’ (p = 0.033, d = 0.77, *moderate*) and ‘multi-phase attack’ (p = 0.003, d = 0.87, *moderate*) during losses. Deceleration was greater for ‘multi-phase attack’ (p = 0.002, d = 0.81, *moderate*) and ‘multi-phase defence’ (p = 0.025, d = 0.94, *moderate*) during wins, when compared with ‘single-phase attack’ during losses.

## Discussion

The aim of this study was to evaluate the effect of discrete passage types on locomotor demands in international men’s and women’s rugby sevens and their relationship with match, whilst controlling for the ranking of the opposition team. The mean duration of each passage type was between 10 s (for ‘single-phase attack’ in women, irrespective of match result) and 113 s (for ‘multi-phase attack to defence’ during wins in men). As per the hypothesis, locomotor demands differed across types of passages. In men, irrespective of match result, relative TD ranged from 123 m∙min^-1^ to 174 m∙min^-1^ for ‘multi-phase defence to attack’ and ‘multi-phase attack’, respectively. In women, ‘multi-phase attack’ elicited the lowest relative TD (118 m∙min^-1^), whereas the greatest value (186 m∙min^-1^) was recorded for ‘single-phase defence’. Knowing the locomotor demands associated with different passages of play may help team coaching staff to design and monitor integrated training activities that simultaneously promote match-play specific physical, technical, and tactical adaptations. Moreover, this information may be useful to help prepare for specific matches, such as occasions when a team might expect to spend less time in possession against a higher-ranking opponent.

For men and women, relative TD during each passage type substantially exceeded values that have been reported across a whole-match [5,7,11]. This stands to reason, given that estimates of whole-match demands typically also include periods for which the ball is out of play and thus underestimate the running demands associated with ball in play time. As the ball may only be ‘live’ for less than 50% of an international rugby sevens match [11], whole-match averages have limited utility for prescribing exercise intensity during specific training drills. Similarly, taking a WCS approach does not involve consideration of the context in which the most demanding periods of play occurred. Each passage type (i.e., single or multi-phase; attack, defence, or transition) requires execution of distinct movement patterns and tactical strategies, and coaching in rugby sevens often involves understanding the behaviours inherent within each passage type The findings of the current study may be used to help inform the design of training activities that develop the necessary technical and tactical skills associated with different within-match objectives, whilst also being conducted at a ‘speed’ that reflects periods of actual match-play. This approach is in keeping with the principles of tactical periodisation and advanced skill acquisition [18].

For men, the lowest relative TD and HSR values were generally recorded for ‘single-phase defence’ during wins. Indeed, relative TD during ‘single-phase defence’ was significantly lower for wins compared with the same passage during losses, with HSR following the same trend. Tackle completion and executing turnovers are defensive characteristics of successful rugby sevens teams [17,23]. For example, winning opposition teams may have more effectively moved the ball into space within the first phase, thus requiring the reference team to cover ground to defend the gain line. The winning teams appeared to require less movement intensity during single-phase defences, which may indicate a more limited use energetic resources during shorter defensive periods. Limiting movement demands during single-phase defence could be facilitated by superior field positioning or other tactical factors such as effective line spacing, and carries the advantage of preserving energy for other passages of the match.

Male players recorded higher relative TD during ‘multi-phase attack’ for wins compared with the same passages during losses. ‘Multi-phase attack’ during wins also appeared to elicit the greatest relative HSR demands of any passage type. Notably, multi-phase attacking passages lasted 37 s during wins, compared with 29 s during losses. Whilst this study cannot determine whether possession was retained through passing or via rucks, these periods naturally reduce the opportunity to expend energy during defensive passages. Taken together, the higher locomotor responses observed for these passages during wins could be attributable to the reference team being more successful in gaining ground when attacking in matches that they ultimately won compared with occasions in which they lost. More successful international rugby sevens teams typically make more entries into the opposition’s 22 m zone (i.e., the area of the pitch within 22 m from the try line and goal posts) per match [23] and are able to retain possession for longer with limited passing and fewer rucks and mauls than their less successful counterparts [17]. Indeed, attacking passages that are less frequently slowed down by successful opposition tackles may give players more opportunity to advance up the pitch within a given time period. Whilst it is unclear whether a higher ‘pace’ of attacking phases is a cause or consequence of team success, it may be suggested that increasing running ‘intensity’ and speed of ball recycling whilst in possession may improve the chances of winning during rugby sevens match-play. Future research should include analysis of the number of contacts, breakdowns, and other technical actions during these different types of passages, to provide a more complete picture of the characteristics of successful teams or players.

Interestingly, the same patterns were not observed in the women’s data. For women, higher relative TD demands were generally observed during ‘single-phase defence’ (wins and losses; 170 to 186 m∙min^-1^), ‘single-phase attack’ (wins; 169 m∙min^-1^), and ‘multi-phase defence’ (losses; 169 m∙min^-1^). The greatest relative HSR was observed for ‘single-phase defence’ during losses (69 m∙min^-1^). Whilst such disparities are difficult to reconcile, data from international 15-a-side rugby union indicates that successful men’s and women’s teams may adopt different attacking and defensive strategies [24]. Acknowledging that the specific tactics will differ from those employed in the 15-a-side game, the current findings may indicate similar tactical discrepancies in international rugby sevens and highlight the need for training prescription to be sex-specific. Notably, whilst women appeared to have longer multi-phase attack and transition passages during losses when compared with the men’s data, this was not the case during wins. Although findings are largely equivocal [12], some existing research has observed greater whole-match locomotor demands during losses, compared with wins [5]. Such observations have been attributed to greater efficiency of winning teams. The findings of the current study may indicate that losing teams experience generally greater demands during defensive passages but conduct their attacking passages at a ‘slower’ pace (i.e., lower relative running intensity).

This study did not include playing position in its analysis. That said, although backs may achieve higher maximal speeds and forwards accumulate more TD during a match [2,11], the physical profiles of rugby sevens athletes are substantially more similar than their 15-a-side counterparts [2,5]. Similarly, rugby sevens permits ‘rolling’ substitutions, whereby players may re-enter the pitch having previously been replaced. Whilst substitutes have been reported to exceed the relative TD and HSR responses of those players who start a match on the pitch [2,11,12], substitutes are often exposed to similar durations of match-play as starting players and thus relative running responses are more similar than those observed in longer duration team sports such as 15-a-side rugby. Therefore, it is recommended that research includes data from substitute players alongside data from those individuals who started a match on the pitch [2].

It should be noted that the same 5 m∙s^-1^ speed threshold was used to define HSR for both men and women in the current study. Whilst running at this speed may not reflect the same level of ‘effort’ for men as it does for women, this study was based on kinematic variables to inform training specificity according to match locomotor activities, rather than determining ’load’ per individual. Moreover, men’s and women’s data were analysed separately as opposed to comparisons being drawn between them. Further work implementing sex-specific thresholds would be useful by considering domains and thresholds more appropriate to female physiology when seeking to assess training magnitude for this population.

Like in many other team sports, reductions in locomotor responses have been observed between halves of rugby sevens matches [2,13]. Similarly, field position might plausibly influence the tactics employed during the different passages of play and thereby affect the locomotor demands of any given passage. For example, greater relative running intensities were reported when Australian Football players attacked from the back half of the field compared with the front half [16]. Future research should consider additional contextual factors such as field position and timing of when the passage occurred, to help add further training specificity. Although this study provides novel data to outline the locomotor demands of different passages of international men’s and women’s rugby seven’s match-play, several important components of physical and technical performance that are beyond the scope of this investigation warrant mention. Contacts are a substantial contributor to the physical loads experienced by rugby sevens players during match-play and training [25,26], whilst several other technical actions are important components of team success [17,23]. As the number and intensity of contacts within a given period could affect the locomotor demands and physiological responses elicited [25,27,28], it will be important for future research to include contacts when quantifying the demands of different passages of play in contact sports. Moreover, true integration of technical/tactical and physical training will require simultaneous consideration of the technical demands of each passage type.

## Conclusions

This study quantified the locomotor demands of different passages of play in international men’s and women’s rugby sevens match-play. Passages in the men’s and women’s matches lasted 10–113 s depending on passage type, whilst locomotor responses were influenced by passage type and match result. Running intensity data for each passage type may inform training drills targeted at developing match-play specific physical, technical, and tactical adaptations. Understanding how passages differ between matches won and lost could also inform team tactics and potentially aid selection planning by matching a player’s fitness characteristics to a team’s tactical decisions.
